# CRISPR is easy: Exposure to *Last Week Tonight* enhances knowledge about gene editing

**DOI:** 10.1371/journal.pone.0306563

**Published:** 2024-10-04

**Authors:** April A. Eichmeier, Michael A. Xenos

**Affiliations:** 1 Department of Emerging Media, University of St. Thomas, St. Paul, Minnesota, United States of America; 2 Department of Life Sciences Communication, University of Wisconsin-Madison, Madison, Wisconsin, United States of America; Philipps-Universitat Marburg, GERMANY

## Abstract

Experts have called for public engagement with the governance of controversial scientific research and discoveries, including CRISPR, the technology that enables gene editing. Though engaging and informing citizens who are not interested in the issue is a challenge, recent studies suggest humor has potential to close interest and knowledge gaps. We tested this potential by exposing individuals (*N* = 303) to one of three videos (an edited clip from *Last Week Tonight*, an edited clip from *60 Minutes*, or control) that contained broadly overlapping facts about gene editing in an online survey. Results show that while exposure to the *Last Week Tonight* clip did not increase attentiveness to the issue of human gene editing among individuals with lower levels of interest in science, exposure to the humorous clip caused a modest improvement in issue knowledge. Positive main effects on perceived knowledge were found for both treatments. More research is needed but findings suggest that the use of humor in science communication offers potential, though perhaps limited, for broadening public engagement with emerging areas of science.

## Introduction

Scholars are giving increased attention to humor as a tool for increasing attentiveness to and knowledge about science. For example, recent research has focused on the potential for humor to foster engagement with science content [1–3], while other research has focused on engaging audiences with polarizing issues like climate change [4], including communicating the certainty of its existence [5–7] and its risks [8, 9]. The present study builds upon this work by focusing on the challenge of engaging individuals who have lower levels of interest in science with the emerging science of human gene editing (HGE).

The stakes for public involvement with the regulation of HGE are high. A report from the National Academies of Sciences, Engineering, and Medicine calls for greater nonexpert involvement in the governance of CRISPR, the technology that enables gene editing, or the altering of DNA, because of its many political and ethical dimensions [10]. Now, the report notes, is the time to address these issues. However, getting timely dialog to happen, when plans could be made for future needs, is a challenge. While there are many citizens who have interest in science and even contribute to it, many of these citizens already have science backgrounds [11]. However, normatively speaking, all citizens should know the facts of HGE and knowledgeably participate in deliberations about it. But there will always be a segment of citizens who just aren’t interested in science.

While no single approach could close all science interest and knowledge gaps, this study advances our knowledge of how citizens who have lower levels of interest in science respond to content that entertains and informs. Past work provides evidence to believe that humorous content such as satire can help engage the uninterested with complex topics: for example, campaign finance is legally complicated, arguably difficult for average citizens to understand, and for some, boring. However, a 2014 study showed that viewing *The Colbert Report* was associated with knowledge about Super PACs and 501(c)(4) organizations more than other sources of news [12]. Similarly, net neutrality, which concerns the regulation of internet traffic, is complicated and uninteresting to many. However, one study found that exposure to *Last Week Tonight* resulted in knowledge gain about the subject [13].

We explore effects of viewing part of an episode of *Last Week Tonight with John Oliver* where the show covered CRISPR, the technology that enables HGE. *Last Week Tonight* is a weekly American late-night show that leverages news for satirical purposes. It has been praised for being informative [14]. During the episode, Oliver discussed what gene editing is and why citizens should care about it: while it could help cure disease, there are many scientific and societal uncertainties that need to be carefully considered. Our theoretical foundation is the gateway hypothesis [15], which explains how content that combines public affairs information with entertainment can help increase attentiveness and knowledge among audiences with lower levels of interest in political topics. Gateway effects have been documented for science issues [4], but the theory merits further investigation. Next, we address a concern that political comedy may foster perceived knowledge rather than factual knowledge. Our experiment (*N* = 303) tested if exposure to a clip from *Last Week Tonight* would result in attentiveness to, knowledge about, and perceived knowledge about the issue of gene editing compared to the control condition, a clip of chef Ina Garten making a coconut cake. Also, because news is a popular source of information about science, we included a condition in the experiment featuring a clip from the weekly news program *60 Minutes*; both experimental conditions provided broadly overlapping facts about gene editing. Together, the conditions provide a descriptive, exploratory comparison to further illustrate the potential effects of the satirical presentation style relative to a familiar reference point.

### Engaging uninterested audiences

Since well before the advent of CRISPR, scholars have studied the potential for entertainment programming–primarily “soft news” in early theoretical work–to broaden engagement with complex public affairs issues [15]. One mechanism for connecting exposure to information delivered within entertaining or humorous content and political knowledge is the “gateway hypothesis,” where soft news serves as a gateway to issue information, as it is delivered in an accessible manner. Soft news attracts and informs viewers through a process of “piggybacking,” whereby information is incorporated into a program while the entertainment purpose is maintained [15]. With this in mind, after exposure to soft news, citizens with low levels of interest in political affairs could become more attentive to an issue. Baum (2003) defines attentiveness as being “*cognizant of an object*, *and selectively process[ing] information about it*,” (p. 11, italics in the original).

In practice, and in studies of the gateway hypothesis, this means a propensity to attend to new information about a given topic when it becomes available, especially among those otherwise not predisposed to do so. A study using a U.S. sample found that, at higher levels of soft news consumption, individuals with low interest in politics were almost as likely to follow news about international conflicts as their high-interest counterparts [15]. Similarly, Xenos and Becker (2009) found that exposure to a clip about the war in Iraq from *The Daily Show* was associated with an increased likelihood of selecting articles about foreign policy in a subsequently encountered information board featuring news articles on a variety of topics [16]. Thus, the effects of the gateway hypothesis are especially pronounced among individuals who have lower levels of interest in political topics. Other studies have found gateway effects in that exposure to late-night television was associated with attention to campaign news [17].

While Baum (2003) focused on soft news, Xenos and Becker (2009) focused on satire. It must be noted that while soft news and satire share an entertainment function, satire is qualitatively distinct from soft news. Not only does satire use humor in service of attacking people or institutions [18], compared to soft news it prioritizes political content, which soft news does not, and the politics are implicit, where soft news politics are explicit [19]. Thus, the effects of exposure to each may differ. Further, differences in effects could be attributable to the fact that viewing satire requires active cognitive engagement to understand the content [20], resulting in a stimulated state.

At the same time, both forms of programming are consistent with core components of the gateway hypothesis, in that scholars argue that individuals consume soft news for the purpose of being entertained [21] and both deliver information in a manner that centers entertainment [19]. The focus on entertainment is evidenced by Oliver himself, who stresses that entertaining people is his goal [22].

### Knowledge gain among uninterested audiences

Having established that exposure to soft news and satire share differences and similarities, both kinds of programming offer issue-relevant information to viewers. Knowledge is considered a precursor to meaningful political engagement as it helps citizens understand the stakes of an issue [23]. In the context of HGE, citizens who have high levels of knowledge are particularly supportive of public input prior to HGE being used on human beings [24].

Besides being accessible and entertaining, soft news and related programming privileges human angles on contemporary topics [25], which could make learning issue-relevant information more enjoyable for individuals with lower levels of interest in political issues. Largely using recognition measures, evidence supports learning from soft news: in terms of knowledge gain, individuals who were inattentive to politics but who watched *The Oprah Winfrey Show* were better able than other inattentive individuals to vote for a candidate that matched their interests [26]. Also, exposure to a *Daily Show* clip enhanced learning about the economy among those with lower levels of political interest [16]. Further, associations between political comedy and factual knowledge gains have been found in other contexts such as learning about current events and political candidates [27], party issue positions [28], primary campaigns [29], Supreme Court candidates [30], as well as a positive effect on internal efficacy concerning understanding politics [31].

### Uninterested audiences and perceived knowledge

Despite positive results, there are doubts that political comedy can inform citizens in a meaningful manner, resulting in the mere perception of knowledge rather than acquisition of knowledge. Studies have shown that exposure to late-night shows may enable recognition of information rather than recall of information [28], and there are concerns about whether recognition of information is meaningful for political participation [32]. Recall is a deeper cognitive task than recognition. Hollander (2005) argues that television viewing helps people think they know about a topic (such as political campaigns), which is not the same as knowing. Viewers may experience what has been called the illusion of knowing phenomenon, where real knowledge and perceived knowledge do not align [33].

Satire may be less effective than hard news for imparting the kind of facts that matter most. Thus, similar to above, exposure to satire and hard news could have different effects. Some scholars consider “hard” news to be the gold standard of providing political information, as scholars have conceptualized hard news as being politically relevant, in that “political relevant” information includes information that is useful to developing policy (e.g. information about laws and the subsequent societal impacts). Hard news is focused on thematic delivery of information (e.g. placing current events in a larger context) and is impersonal and unemotional. Soft news focuses on information that is less politically relevant, on individuals and individual stories, and is “personal and emotional” [25]. However, Reinemann et al. (2012) acknowledge that news can be harder or softer, suggesting a continuum rather than a category.

Even if soft news and satire feature emotional content, this does not mean that satire lacks political relevance. The political relevance of *Last Week Tonight* is demonstrated by its pointed criticism of its subjects [18]. For instance, one episode concerned the potential for the Federal Communications Commission (FCC) regarding changes to allow internet service providers to manipulate internet speed. In a move that exemplifies political relevance, Oliver called on viewers to comment on the rule online, and the FCC website crashed [34].

Regardless, the news may still be better at communicating facts. One study found that exposure to hard news enabled recall of information about the nomination process of Supreme Court Justice John Roberts, his issue positions, and some personal information, whereas political comedy enabled recall of only personal information [30]. Similar studies show that news has a positive association to possession of political facts whereas late-night programming has a weaker or negative effect [32, 35]. Another concern is misinterpretation of information found in satire, as it is filtered through a partisan lens [36]; one study showed that more than 10% of study participants claimed information from satire to be true [37].

Illusions of knowledge complicate healthy deliberation. Scholars have found that confidence in knowledge is associated with political discussion just as much as possessing actual knowledge [38] and that extreme attitudes toward genetic technologies are associated with more confidence in knowledge even if the confidence is not merited [39]. Being confident enough to engage with issues might be positive from an engagement standpoint, but not from a deliberative standpoint. Thus, perceived knowledge could lead to less-informed individuals making arguments that have a nonfactual basis, to the detriment of deliberation [33]. As a result, using humor to communicate might be counterproductive if perceived knowledge gains outstrip actual knowledge gain.

## Hypotheses

We address whether exposure to political comedy spurs attentiveness and stimulates gains in factual knowledge about gene editing, and we also seek to address whether exposure to political comedy could merely enable perceived rather than real (factual) knowledge. The gateway hypothesis stipulates that exposure to soft news should be associated with subsequent information seeking about political issues, and the effect should be pronounced at lower levels of interest. Thus:

*H1a*: *Those exposed to the political comedy condition will be more attentive to the issue of HGE after exposure than those exposed to control*.
*H1b*: *The effect of exposure to political comedy on attentiveness to the issue of HGE will be stronger for those with lower levels of interest*.
There is evidence that exposure to political comedy results in knowledge gain, particularly at lower levels of interest. Thus:
*H2a*: *Those exposed to the political comedy condition will show knowledge gain about the issue of HGE after exposure than those exposed to control*.
*H2b*: *The effect of political comedy on knowledge gain about the issue HGE will be stronger for those with lower levels of interest*.

However, critics have argued that exposure to political comedy might contribute to the perception that one is informed regardless of how much information is obtained, and this effect may be pronounced for those with lower levels of interest.

*H3*: *Exposure to the political comedy condition will be positively associated with perceived knowledge*.

## Data and methods

The software program G*Power was used to determine the sample size needed. G*Power is a statistical program that is designed for power analyses [40]. The following steps were taken to arrive at a sample size needed. First, we specified that an F test was needed because an F test is appropriate for multiple linear regression, specifying Cohen’s f2 as effect size. A challenge is that studies in this body of scholarship have a wide range of effect sizes. Thus, to be cautious, .07 was chosen. Next, we specified that the test was a priori. Then, we specified up to 10 predictors, in part because there were other studies that could come from the same dataset. Power was assigned as .9. This resulted in a sample size of 303.

Responses (N = 303) were collected via Amazon’s MTurk platform from 11 to 18 May 2020. MTurk is an online labor market where individuals can receive compensation for various tasks, including taking social science surveys. MTurk has advantages, like being fast and accessible, but also disadvantages, like issues with quality control [41]. To help ensure quality, an attention check was embedded in the knowledge scale, where participants were instructed to indicate a specific response. The knowledge scale appeared in the latter half of the survey. Institutional Review Board approval from the University of Wisconsin-Madison was received prior to data collection. Participants were first presented with a screen describing the nature of the study and given the option to participate or end the survey; participants clicked on a link to give their written consent. After consent, the study began. The average completion time was 14 minutes. Respondents were paid $2.50 to complete the survey.

We could not measure knowledge about gene editing before the stimuli without priming the respondents that knowledge could be tested. Thus, this study was a post-test only control group design, which permits comparison between groups and controls for a number of threats to internal validity [42]. In this between-subjects study, participants were randomly exposed to one of three conditions inside the survey platform: a political comedy video, a news video, or a control video. The political comedy video was from the episode of *Last Week Tonight* that aired on June 30, 2018, where John Oliver explains gene editing with his usual, comedic delivery style. The news video was from an episode of *60 Minutes* which aired on April 26, 2018. In this video, journalist Bill Whitaker delivers a story about gene editing in traditional journalistic style. We also used a clip on the *60 Minutes* video that did not appear in the broadcast [43]. The clip was from the *60 Minutes* YouTube channel and features a scientist giving warnings about possible negative effects of gene editing.

Though both clips featured facts, the primary difference concerns presentation. Baumgartner and Morris (2006) compared *The Daily Show* and the *CBS Evening News*, noting that, “the primary difference between them is that The Daily Show focuses on generating humor and sarcasm, whereas CBS Evening News focuses on presenting serious television news” (p. 347). We had a similar situation: the *60 Minutes* clip is journalism, with a calm delivery by Whitaker. The *Last Week Tonight* clip is political comedy, where Oliver goes between quick delivery of jokes and seriousness. However, while the clips differed in presentation, both clips showed how gene editing could benefit humanity in terms of curing disease: Whitaker spoke with a scientist about how gene editing could cure thousands of diseases, and Oliver showed a news segment that showed a baby who benefitted from a gene-editing treatment. Both clips also featured concerns about the ethics of gene editing: Whitaker pressed a scientist about the ethics of editing the genetics of embryos, and Oliver showed Jennifer Doudna, a scientist credited with the development of CRISPR, discussing a CRISPR-related nightmare.

The experimental condition videos were edited to focus on the facts and for length; each was around four minutes long. Both conditions shared the following pieces of information: one, CRISPR is a tool that helps scientists change DNA easily; two, CRISPR could be used to help cure diseases in humans; and three, CRISPR treatments are not ready for widespread use on humans today. The control condition was a cooking video, and featured Ina Garten baking a coconut cake. Note that these facts are domain specific as they pertain only to gene editing. Knowledge that is situation-specific is most suitable for political engagement [23]. Respondents were able to advance the video near the four-minute mark.

### Dependent variables

Attentiveness was proxied by exposing participants to a screen that gave them an option of accessing one of three news articles, an article from the *New York Times* about the ethics of gene editing (chosen by 52.15% of the respondents), an article from the *New York Times* about Saturn’s moons (chosen by 34.32%), or an article from *Business Insider* about celebrity wealth (chosen by 13.53%). The order of the articles was randomized to mitigate order effects. This screen came after viewing the stimulus and was modeled on the operationalization of attentiveness in prior research on the gateway hypothesis in a political context (Xenos and Becker 2009). The articles did not have source information at the point of selection, as partisan cues can affect the selection process [44]. While it was an option to ask study participants directly whether they had become more attentive to the issue of HGE, in a time-limited experimental setting, article selection makes sense as a measure of attentiveness. Also, the behavioral measure has the advantage over self-report as we were able to observe behavior rather than relying on a report of follow up information-seeking. While there are scholarly debates regarding social desirability and reliability of self-report data, many consider observational measurements as ground truth indicators of purposeful media consumption [45].

Video-specific knowledge was measured with three questions regarding the facts from the clip mentioned above. On the scale, one was “definitely false” and four was “definitely true.” There was also an option to answer, “don’t know.” The don’t know answers were coded as missing. The questions were converted to dichotomous true/false measures. Each item was run individually in the analysis to measure where learning might occur. To keep the experiment short and to discourage searching for the answers online, respondents were given 15 seconds to answer each question before the screen automatically advanced.

Perceived knowledge was measured with the question, “How well informed would you say you are about gene editing?” where one was “Not at all informed” and five was “Very informed” (M = 2.21, SD = .81). This measure appeared after the videos and articles but before the knowledge battery.

### Independent variables

Interest in scientific research and discoveries was measured with an adapted question from the science curiosity scale, which was developed to reduce social desirability by making it seem like a marketing survey: participants are asked to indicate their interest in a variety of topics, from “interest in scientific research and discoveries” (the key variable in this study) to other topics such as “entertainment and celebrities” [46]. The question read, “Here are some topics that some people are interested in, and some people are not interested in. For each topic, please indicate **how interested** you are in that topic” where zero was, “not at all interested” and three was, “very interested” (M = 2.07, SD = .88) (Bold is in the original science curiosity scale.) The order of the response options was randomized to mitigate order effects.

Because it was possible that information about gene editing could be obtained through the news, and previous research identifies science news attention as a predictor of HGE knowledge [47], news was measured as usage of the following three media: newspapers (M = 3.43, SD = 1.46), television (M = 4.02, SD = 1.20), and social media (M = 3.75, SD = 1.46). The order of the questions was randomized to mitigate order effects.

Several demographic variables were also included as control variables because they could be associated with the dependent variables. Age was measured with the question, “What year were you born?” (M = 45.58, SD = 11.76). Gender was measured with the question, “Please indicate your gender” where answers were male, female, my gender is not listed here, and prefer not to say; males comprised 46.9% of the sample; this variable was dummy coded in the analysis, with male being 1 (N = 141) and else (female, other) being 0 (N = 161). Education was measured with the question, “Please indicate your highest level of education” (High School, College, etc.) (M = 2.46, “some college”, SD = 1.25).

## Results

Table 1 shows the means and standard deviations of the variables across each condition. An ANOVA test for differences between groups was nonsignificant (.23), indicating successful randomization of participants into the conditions.

**Table 1:** Means and standard deviations by condition.

	Last Week Tonight	60 Minutes	Control
**Age**	44.63 (12.52)	42.77 (10.41)	43.31 (12.28)
***N* Male**	52	47	43
**Education**	4.23 (1.25)	4.41 (1.2)	4.15 (1.30)
**Interest in science**	2.13 (.81)	2.1 (.90)	1.99 (.93)
**Newspaper**	3.55 (1.42)	3.35 (1.50)	3.43 (1.46)
**Television**	4.01 (1.18)	4.00 (1.24)	4.02 (1.20)
**Social media**	3.83 (1.48)	3.50 (1.25)	3.75 (1.48)
***N* chose HGE article**	54 (51.9%)	57 (55.3%)	47 (49.0%)
***N* correct–easy**	87 (83.7%)	93 (90.3%)	69 (71.9%)
***N* correct–cures**	98 (94.2%)	96 (93.2%)	65 (67.7%)
***N* correct–wide use**	82 (78.8%)	87 (95.2%)	63 (65.6%)
**Perceived knowledge**	2.30 (.76)	2.41 (.82)	1.89 (.75)
***N total per condition***	104	103	96

Standard deviation is in parentheses.

The analysis was conducted using ordinary least squares (OLS) because OLS permits studies that accommodate how levels of a personal characteristic (e.g. interest in scientific research and discoveries) and an experimental condition jointly affect a dependent variable (e.g. perceived knowledge); variables are entered in blocks in order of assumed causality [48].

SPPS version 29 was used to estimate the models. Outliers were detected by examining the standardized residuals, where values in excess of +2 and -2 were considered outliers; there were no outliers in models 2, 3, and 4 and 13 outliers in model 4. Influential points were measured by Cook’s distance, where 1 was considered the threshold for an influential point; there were no influential points in any of the models. Thus, outliers were kept for analysis. Models were assessed for collinearity using the main effect variables; no issues with collinearity were detected (all models had acceptable VIF and tolerance scores). However, given that this study focuses on interactive effects, some collinearity is expected. To address this issue, all results are reported in blocks, thus, the main effects shown below represent an independent variable’s relationship to the dependent variable without the interactions; and the interaction variables represent effects in addition to the main effect variables.

To test *H1a* and *H1b* we specified a logistic regression model predicting the likelihood of participants selecting the article about gene editing as a function of relevant control variables, assignment to each of the experimental conditions, interest in science, and interaction terms designed to probe the extent to which the effects of exposure to either the *Last Week Tonight* or *60 Minutes* clips are moderated by science interest. If exposure to the political comedy condition has an effect consistent with the gateway hypothesis, it should interact with interest in scientific research and discoveries, and lower levels of interest should be associated with a higher probability of accessing information than would normally be expected.

Table 2 shows the results. The Pseudo R^2^ for the full model was 6.8%. In the final model, no variables were significantly associated with accessing the article. The interactions were nonsignificant. Thus, *H1a* and *H1b* were not supported. Exposure to political comedy was not associated with higher levels of attentiveness at any level of interest in scientific research and discoveries.

**Table 2:** Logistic regression analyses for the probability of choosing the gene editing article (model 1).

Block	Odds ratio	95% CI	Sig.
**1: Demographics**			
** Age**	1.02	1.00, 1.04	.06
** Male**	1.32	.68, 1.80	.61
** Education**	.88	.68, 1.01	.17
**R** ^**2**^	3.1%		
**2: Interest**			
**Interest in scientific research and discoveries**	1.08	.90, 1.54	.22
**R** ^**2**^	2.8		
**3: News**			
**Newspaper**	1.09	.92, 1.29	.30
**Television**	1.11	.90, 1.36	.33
**Social media**	1.12	.96, 1.31	.16
**R** ^**2**^	4.4%		
**4: Conditions**			
**Sixty Minutes**	.69	.38, 1.22	.20
**Last Week Tonight**	.90	.51, 1.59	.71
**R** ^**2**^	5.0%		
**5: Interactions**			
60 Minutes*interest	1.02	.54, 1.91	.96
Last Week Tonight*interest	.82	.41, 1.61	.56
** R** ^**2**^	6.8%		
** *N***	303		
***N chose article***	158		
***df***	8		
**X** ^**2**^	15.85		.15

Degrees of freedom and X^2^ are from the final model. R^2^ is Nagelkerke R^2^.

An alternative specification that excludes the demographic controls and news variables can be found in the supplementary materials. The substantive interpretations of both sets of models are the same.

To test *H2a* and *H2b*, we specified a series of logistic regression models following the same structure as those used to test *H1a* and *H1b*, with individual items. Table 3 shows the results of these analyses. As with the previous model, if exposure to the political comedy condition has an effect consistent with the gateway hypothesis, it should interact with interest in scientific research or discoveries, and lower levels of interest should be associated with a gain in knowledge.

**Table 3:** Probability of choosing the correct answer from the clip (models 2 through 4).

Block	Easy			Cures			Wide Use		
	Odds	95% CI	Sig.	Odds	95% CI	Sig.	Odds	95% CI	Sig.
**1: Demo.**									
** Age**	1.01	.98, 1.04	.58	.98	.94, 1.02	.43	.99	.96, 1.01	.36
** Male**	.92	.42, 1.97	.82	1.15	.43, 3.06	.78	1.32	.69, 2.53	.41
** Educ.**	.92	.68, 1.25	.59	1.23	.86, 1.76	.27	1.13	.88, 1.45	.34
**R** ^**2**^	< .01%			1.7%			1.2%		
**2: Interest**									
**Interest**	1.06	.69, 1.61	.80	2.01	1.18, 3.41	.01	1.30	.91, 1.85	.16
**R** ^**2**^	.01%			7.6%			2.3%		
**3: News**									
**Newspaper**	1.41	1.08, 1.85	.01	1.39	.99, 1.95	.06	1.03	.82, 1.29	.82
**Television**	.88	.62, 1.25	.48	1.31	.88, 1.96	.19	1.05	.79, 1.39	.75
**Social media**	1.11	.87, 1.42	.39	1.05	.76, 1.44	.79	.83	.65, 1.05	.12
**R** ^**2**^	5.8%			12.9%			3.8%		
**4: Cond.**									
** 60 Min.**	.38	.13, 1.07	.07	.24	.07, .83	.02	.46	.20, 1.05	.07
** LWT**	1.04	.43, 2.51	.94	.25	.07, .89	.03	.74	.35, 1.54	.42
**R** ^**2**^	4.7%			19.3%			5.9%		
**5: Inter.**									
60 Min.*Int.	.65	.21, 2.03	.46	1.50	.40, 5.61	.55	1.09	.46, 2.60	.85
LWT*Int.	.32	.11, .92	.04	2.26	.50, 10.33	.29	.62	.26, 1.49	.28
**R** ^**2**^	6.3%			20.4%			5.9%		
***N***	281			278			280		
***N correct***	249			259			232		
***df***	8			8			8		
**X** ^**2**^	11.46		.08	23.15		.02	10.04		.35

Degrees of freedom and X^2^ are the final block. R^2^ is Nagelkerke R^2^.

Regarding answering the question about the ease of gene editing, the Pseudo R^2^ for the full model was 6.3%. In the final model, newspaper use (1.41, p = .01) and the interaction between interest in scientific research and discoveries and the *Last Week Tonight* condition was significant (.32, *p* = .04).

Given this result, we used the SPSS PROCESS macro [49] model 1 to probe the significant interaction using the Johnson-Neyman technique. We specified that Y was the “easy” question, X was *Last Week Tonight*, and the moderator (W) was interest. All other variables from the model above were included as covariates. Though the results showed that the interaction findings were not significant by conventional standards, there is evidence, if modest, that at the lowest level of interest (0 level of interest, p = .08), information-laden entertainment content can exert an equalizing effect, producing expected values for low and high interest participants that are effectively indistinguishable.

**Fig 1:**
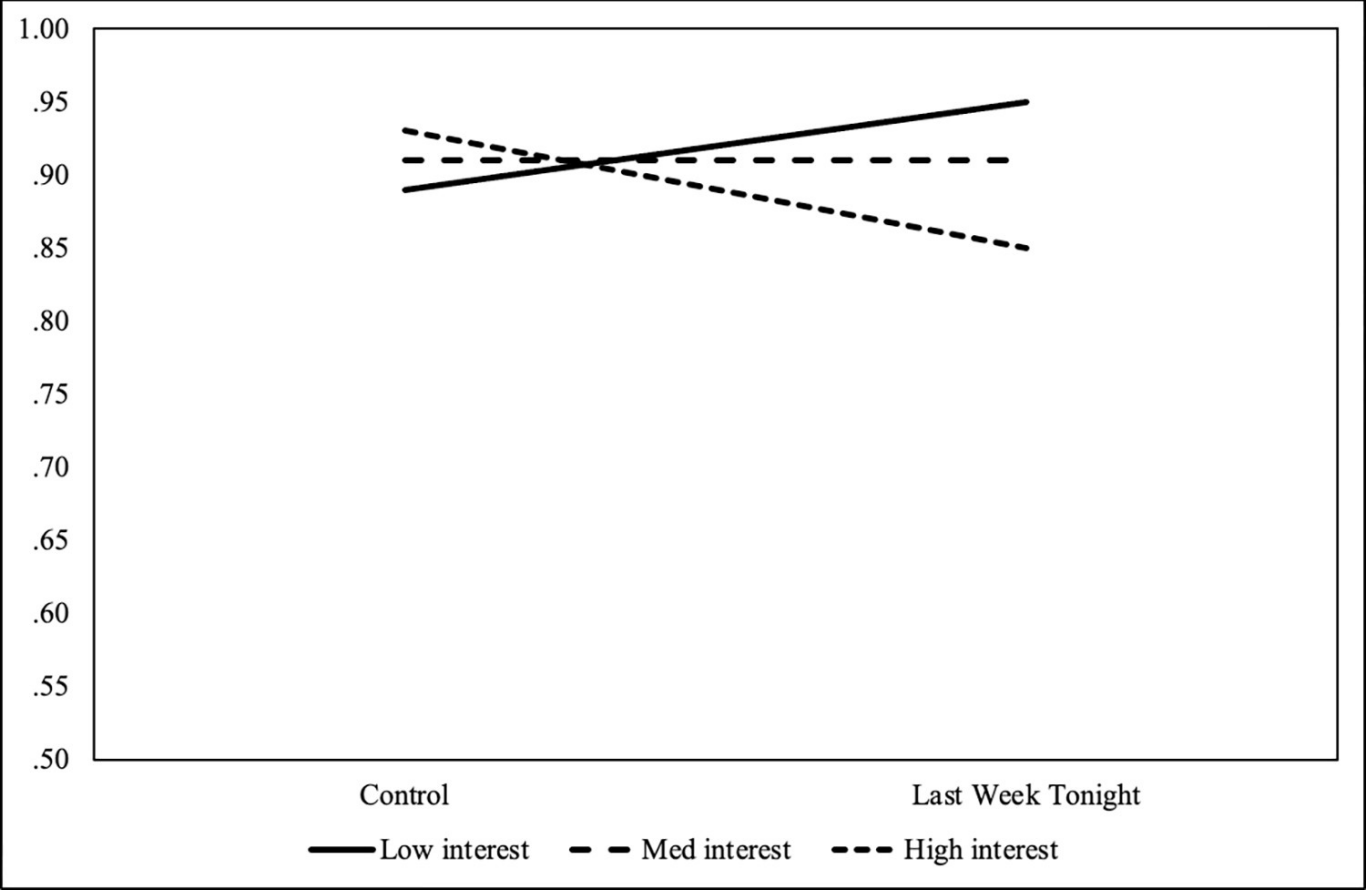
Last Week Tonight compared to control regarding the correct answer to CRISPR’s ease of use.

Next, we again used SPSS PROCESS macro model 1 to compare *Last Week Tonight* to control and to plot *Last Week Tonight* compared only to the control condition. Levels of interest were conditioned on -1 standard deviation (low interest), +1 standard deviation (high interest) from the mean (medium interest). Comparing the control condition and *Last Week Tonight*, at the lowest level of interest, there was a .89 and .95 probability of answering the question correctly, respectively. Notably, comparing the control condition and the *Last Week Tonight* condition at the highest levels of interest, there was a .93 and .85 probability, respectively. See Fig 1.

**Fig 2:**
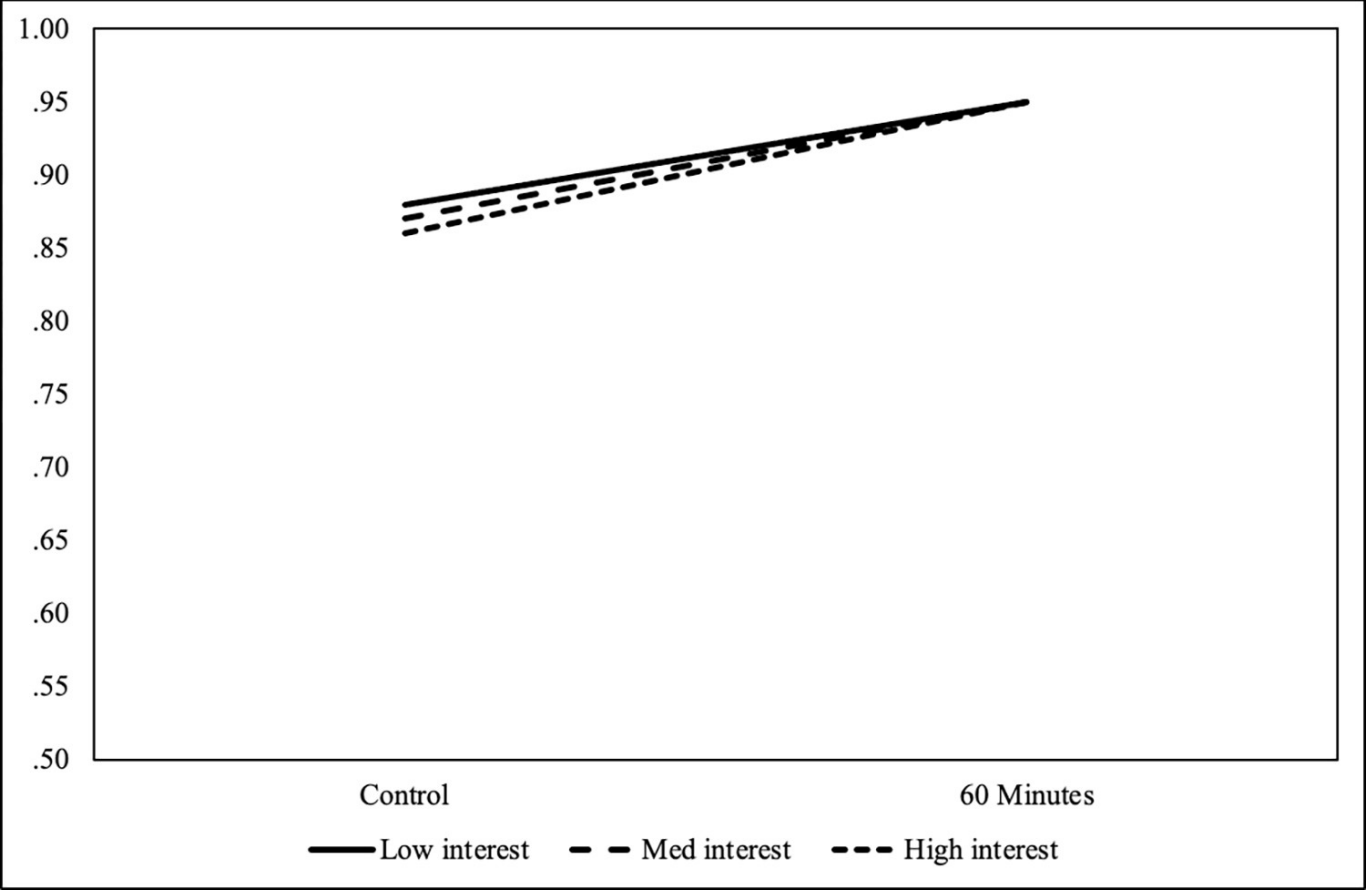
60 minutes compared to control regarding the correct answer to CRISPR’s ease of use.

Though we did not find a significant result for the *60 Minutes* condition, we offer it to show a comparison. Using the same specifications as above but X was specified as *60 Minutes*. Comparing the control condition and *60 Minutes*, at the lowest level of interest, there was a .88 and .95 probability of answering the question correctly, respectively. See Fig 2.

In the *Last Week Tonight* condition, participants with the lowest levels of science interest showed distinct gains in knowledge from exposure to the condition, whereas there seem to be, if anything, negative gains from exposure for those with higher levels of interest.

For answering the question about whether gene editing could mean cures for disease, the Pseudo R^2^ for the full model was 20.4%. In the final model, interest was positively and significantly related to answering correctly (2.01, *p* = .01) as was the *60 Minutes* condition (.24, *p* = .02) and the *Last Week Tonight* condition (.25, *p* = .03). The interactions were nonsignificant. Regarding answering the question about whether gene editing was ready for widespread use, the Pseudo R^2^ for the full model was 5.9%. All independent variables had a nonsignificant relationship to answering the question correctly. The interactions were nonsignificant. Thus, *H2a* and *H2b* receive mixed support.

An alternative specification that excludes the demographic controls and news variables can be found in the supplementary materials. The substantive interpretations of both sets of models are the same; with or without controls, we find mixed support for *H2a* and *H2b*.

As noted earlier, we also sought to assess the possibility raised by critics of the gateway hypothesis, and the educative potential of political entertainment programming more broadly, by exploring possible effects on perceived rather than gains in actual knowledge. Though the above results already rule out the possibility of a “mere” perceived knowledge effect, it is still useful to examine self-reported perceived knowledge as an outcome variable. Table 4 shows the results.

**Table 4:** Regression analyses showing entries by block for perceived knowledge (model 5).

	B	95% CI	SE	β	Sig.
**1: Demographics**					
** Age**	-.01	-.01, .00	.00	-.08	.16
** Gender (Male = 1)**	.13	-.05, .32	.09	.08	.15
** Education**	-.03	-.10, .05	.04	-.04	.49
**Inc. Adjusted R**^**2**^ **(%)**	.3%				
**R**^**2**^ **Change**	.01				.29
**2: Interest**					
**Interest in scientific research and discoveries**	.16	.06, .27	.05	.18	.01
**Inc. Adjusted R**^**2**^ **(%)**	3.8%				
**R**^**2**^ **Change**	.04				< .001
**3: News**					
**Newspaper**	-.01	-.07, .05	.03	-.02	.78
**Television**	.04	-.04, .12	.04	.06	.32
**Social media**	.01	-.05, .07	.03	.01	.83
**Inc. Adjusted *R***^**2**^ **(%)**	3.1%				
**R**^**2**^ **Change**	.00				.83
**4: Conditions**					
** Sixty Minutes**	.52	.30, .74	.11	.31	< .001
** Last Week Tonight**	.40	.18, .61	.11	.23	< .001
**Inc. Adjusted R**^**2**^ **(%)**	9.9%				
**R**^**2**^ **Change**	.07				< .001
**5: Interactions**					
60 Minutes*Interest				.06	.70
Last Week Tonight*Interest				.16	.32
**Inc. Adjusted R**^**2**^ **(%)**	9.9%				
**R**^**2**^ **Change**	.01				.38
**N = 303**					
**Total *R***^**2**^ **(%)**	13.3%				

Cell entries are final standardized regression coefficients for blocks 1 to 4, and before-entry standardized regression coefficients for block 5.

Results shown in Table 4 suggest that exposure to both the *Last Week Tonight* and *60 Minutes* clips stimulated gains in perceived knowledge, though there is no evidence of moderation of these effects by levels of interest in science. The full model explains 13.3% of the variance in perceived knowledge. Two variables were significantly associated with the dependent variable: interest in scientific research and discoveries (β = .18, *p* = .01) and the experimental conditions (β = .31, *p* = < .001; β = .23, *p* = < .001). The interactions were nonsignificant. Thus, support is mixed for *H3* in that exposure to political comedy did stimulate higher levels of perceived knowledge, though this effect was also found for the *60 Minutes* condition and was not conditional on lower levels of interest in scientific research and discoveries.

An alternative specification that excludes the demographic controls and news variables can be found in the supplementary materials. In both sets of results, *H3* has mixed support.

## Discussion and conclusions

This study tested whether exposure to a political comedy clip would result in increased attentiveness, knowledge, and perceived knowledge about HGE among individuals with lower levels of interest in science. The results provide mixed support for the gateway hypothesis as applied to an emerging science issue, human gene editing. The first analysis did not support the proposition that political comedy increases attentiveness and spurs information seeking. The second analysis provided modest support to the proposition that political comedy enhances knowledge acquisition amongst the less interested. The third found a significant, positive main effect of the humorous clip on perceived knowledge, but no variation in this effect based on interest in science.

This study shows that there is potential for political comedy and other forms of entertaining or humorous content to be a tool engaging for audiences who aren’t already interested in scientific topics. But the results are mixed and stand somewhat in contrast to patterns of findings in other issue domains. While desirable from an engagement standpoint, results from this case suggest caution in viewing humor as a dependable strategy for engaging and informing less interested audiences about controversial science. While the results show some potential in humor as a strategy to get the less-interested at least part way there, it would be a stretch to interpret these findings as suggesting that political comedy is the key to closing interest and knowledge gaps and enhancing deliberation about controversial science. Moreover, the clearer signal in these data for *perceived* issue knowledge provided by the positive and significant main effect for comedy exposure lends some support to concerns that humorous presentation may merely promote the illusion of knowledge gain for most viewers. At the same time, the effect was also found from exposure to the *60 Minutes* condition. While our findings support concerns about comedy, these effects are comparable to those observed for news content.

Before further discussion of the results and implications of the findings, it is important to consider limitations to this study. First, this experiment was designed to manipulate only one key variable: exposure to a political comedy program or a news program. Experiments have a high degree of internal validity, and it can be claimed that exposure to the political comedy caused some knowledge gain. But, as with most experiments exploring potential knowledge-gain effects flowing from humor, this experiment is not reflective of a real media environment. Under normal conditions, viewers can multiscreen, switch programs, etc., and respondents were placed in conditions that they did not choose. At the same time, a primary goal of an experiment is for the treatment to affect the independent variable [50], and this study, if modestly, increased knowledge.

Another concern is that not all forms of variability can be controlled because *60 Minutes* and *Last Week Tonight* are qualitatively different. As mentioned above, experiments have high internal validity, but the variability in the conditions means perfect internal validity is not possible. In our design, this reflects a calculated trade-off between the realism of actual television content, and comparability of the two stimuli, which we optimized as much as possible through careful editing of the clips for informational content and length. As discussed above, the two clips differ in terms of presentation style, such as the presence of jokes in *Last Week Tonight* and the lack them in *60 Minutes*. At the same time, one study showed that *Last Week Tonight* in the 2017 season featured similar proportions of facts and jokes over the course of the season [51], thus, the presence of jokes does not necessarily mean diminished factual value. Finally, as is discussed below, the *Last Week Tonight* features an analogy regarding the ease of CRISPR, which could confound results. However, the use of analogies is an area for future research.

Next, given that past work in this line of research shows consistent results, and though the sample size was sufficient per the power analysis, it is still possible that some results went undetected. Further, the results apply only in the U.S. context, and are limited to the specific issue domain of HGE technologies. Future research aimed at assessing the potential for humor to enhance public engagement with science should pursue variations in issue context.

Returning to the results, none of the conditions predicted accessing the *New York Times* article about gene editing. Though demand characteristics may explain why so many individuals chose to read the article about gene editing, it may also be due to the historical context in which the survey was fielded. Scores on this variable range from zero to three, but 73% were either two or three on the scale, which means the results might not show some effects that would otherwise be detectable. It could be a fluke, it could be that individuals who take MTurk samples are prone to interest in science, but it could also be that there was increased interest in scientific research and discoveries at the time of fielding, as science was in the daily headlines. Use of this scale in another study (reference here) which was fielded in 2018 on a larger, more representative sample showed that interest was a one-half point lower.

While the gateway hypothesis assumes that there is subsequent information seeking about issues after exposure to soft news, particularly among the less interested, future research should address whether such individuals might be motivated to seek political comedy as a primary source of information rather than standard hard news. There is evidence that lower-interest audiences will choose satirical news clips over standard news clips [52], and there is some evidence that this may be occurring for HGE: when the show aired in June of 2018, there was a spike in YouTube searches for “John Oliver gene editing” and a smaller spike around the time that a Chinese scientist announced the birth of gene-edited twins [53], suggesting that individuals sought the video for information about gene editing.

In terms of knowledge gain, we found patterns consistent with the gateway hypothesis for a key piece of information relevant to the broader issues surrounding HGE. When considered alongside the positive main effect of comedy viewing on perceived knowledge, however, these results suggest that future science communication research may benefit from deeper attention to debates surrounding the effects of exposure to “infotainment” programming in other disciplines.

A proposition in the gateway hypothesis is that soft news enables understanding by being accessible. In both experimental conditions, the fact that CRISPR makes editing DNA easy is stated: in the *60 Minutes* clip, Bill Whitaker states this in his introduction; the *Last Week Tonight* clip features geneticist and CRISPR pioneer Jennifer Doudna comparing it to “cutting and pasting” in word processing. But there is an important distinction to make. Whitaker directly states that CRISPR enables easy editing, but the *Last Week Tonight* clip featured Doudna making an analogy. It is possible that the lower-interest individuals keyed into the word processing analogy, which could have resonated with individuals with lower levels of interest in science. Analogies may be particularly effective at communicating complicated topics: Oliver frequently uses analogies for complex topics, like comparing the ability of internet service providers to fairly regulate internet traffic speed with the ability of a dingo to be a good babysitter. The analogy was perhaps outrageous, but effective at demonstrating the principle.

However, the question of whether or not CRISPR is an easy way for scientists to edit DNA is arguably a very simple question, which speaks to the criticism that political comedy only enables learning answers to simple knowledge questions [28]. Thus, the potential of political comedy to inform lower-interest individuals with more complex facts may be marginal. However, future research should address if and under what circumstances political comedy could be most beneficial in terms of knowledge and engagement. For example, *The Colbert Report* did a series about campaign finance, not only a single show. Regarding the other questions from the clip, correctly answering the question regarding how CRISPR could cure diseases could have tapped into existing schema about how science is the source of cures for disease. Future research could separate schema from knowledge gain.

In this study, we have provided a multi-layered set of tests that explore the potential value of humor as a delivery style for information about a complex scientific issue, particularly for individuals with relatively low intrinsic interest in such issues. Specifically, we explored the potential for humor to stimulate positive effects on issue attentiveness, as well as real and perceived knowledge about the issue, with attentiveness and knowledge-gain effects concentrated among participants with lower levels of science interest. While our results include examples of such gateway effects for knowledge gain, there were multiple pieces of information for which the hypothesized gateway effect was not observed, despite a positive and significant effect on perceived knowledge. Thus, while providing some additional support for the idea that humor may provide an important tool for science communicators seeking to broaden engagement with science and technology issues, our study simultaneously suggests that we may not be able to rely on humor to close interest and knowledge gaps in all areas. Rather, these results suggest that additional research into the use of humor in science communication is worthwhile, particularly given the potential for humorous communication strategies to create unintended gaps in real versus perceived knowledge.

## Supporting information

S1 File Supporting data. (CSV)

S2 File Syntax. (DOCX)

S3 File Supplemental analyses. (DOCX)
