# Supplementary material for: CRISPR is easy: Exposure to Last Week Tonight enhances knowledge about gene editing
Source: PLoS One. 2024 Oct 4;19(10):e0306563. doi: 10.1371/journal.pone.0306563 (PMC11452040; doi:10.1371/journal.pone.0306563)
Supplement: S2 File — (DOCX) [file pone.0306563.s002.docx]

**SPSS Syntax for CRISPR is easy: Exposure to Last Week Tonight enhances knowledge about gene editing, PLoS, June 2024**

1. **SPSS Syntax for the manuscript.**

*checking for regression assumptions (logistic models)

REGRESSION

/DESCRIPTIVES MEAN STDDEV CORR SIG N

/MISSING PAIRWISE

/STATISTICS COEFF OUTS CI(95) R ANOVA CHANGE COLLIN TOL CHANGE ZPP

/CRITERIA=PIN(.05) POUT(.10)

/NOORIGIN

/DEPENDENT gearti

/METHOD=ENTER age male educ

/METHOD=ENTER sciint

/METHOD=ENTER npaper televis socmdia

/METHOD=ENTER SixtyCD LWTCd

/METHOD=ENTER sixty_int sciint LWT_int

/RESIDUALS DURBIN HISTOGRAM(ZRESID) NORMPROB(ZRESID)

/CASEWISE PLOT(ZRESID) OUTLIERS(3)

/SAVE PRED COOK LEVER SRESID SDRESID.

*DV: choosing the gene editing article.

LOGISTIC REGRESSION VARIABLES gearti

/METHOD=ENTER Age Male educ

/METHOD=ENTER sciint

/METHOD=ENTER npaper televis socmdia

/METHOD=ENTER SixtyCd LWTCd

/METHOD=ENTER sixty_int LWT_int

/SAVE=PRED COOK LEVER DEV SRESID

/PRINT=GOODFIT CI(95)

/CRITERIA=PIN(.05) POUT(.10) ITERATE(20) CUT(.5).

*DV: getting the "CRISPR is easy" question correct.

LOGISTIC REGRESSION VARIABLES easy

/METHOD=ENTER Age Male educ

/METHOD=ENTER sciint

/METHOD=ENTER npaper televis socmdia

/METHOD=ENTER SixtyCd LWTCd

/METHOD=ENTER sixty_int LWT_int

/PRINT=GOODFIT CI(95)

/CRITERIA=PIN(.05) POUT(.10) ITERATE(20) CUT(.5).

*Plotting the interaction LWT vs control

LOGISTIC REGRESSION VARIABLES easy

/METHOD=ENTER Age Male educ

/METHOD=ENTER sciint

/METHOD=ENTER npaper televis socmdia

/METHOD=ENTER SixtyCd LWTCd

/METHOD=ENTER LWT_int

/PRINT=GOODFIT CI(95)

/CRITERIA=PIN(.05) POUT(.10) ITERATE(20) CUT(.5).

*DATA LIST FREE/

LWTCd sciint easy prob .

* BEGIN DATA.

* .00 1.19 2.07 .89

1.00 1.19 2.96 .95

.00 2.07 2.32 .91

1.00 2.07 2.34 .91

.00 2.95 2.57 .93

1.00 2.95 1.71 .85

END DATA.

*Plotting the interaction 60 minutes vs control

LOGISTIC REGRESSION VARIABLES easy

/METHOD=ENTER Age Male educ

/METHOD=ENTER sciint

/METHOD=ENTER npaper televis socmdia

/METHOD=ENTER SixtyCd LWTCd

/METHOD=ENTER sixty_int

/PRINT=GOODFIT CI(95)

/CRITERIA=PIN(.05) POUT(.10) ITERATE(20) CUT(.5).

* DATA LIST FREE/

SixtyCd sciint easy prob .

* BEGIN DATA.

* .00 1.19 1.96 .88

1.00 1.19 2.87 .95

.00 2.07 1.90 .87

1.00 2.07 2.87 .95

.00 2.95 1.83 .86

1.00 2.95 2.88 .95

END DATA.

*DV: getting the "cures" question correct.

LOGISTIC REGRESSION VARIABLES cure

/METHOD=ENTER Age Male educ

/METHOD=ENTER sciint

/METHOD=ENTER npaper televis socmdia

/METHOD=ENTER SixtyCd LWTCd

/METHOD=ENTER sixty_int LWT_int

/PRINT=GOODFIT CI(95)

/CRITERIA=PIN(.05) POUT(.10) ITERATE(20) CUT(.5).

*DV: getting the "wide use" question correct.

LOGISTIC REGRESSION VARIABLES wideuse

/METHOD=ENTER Age Male educ

/METHOD=ENTER sciint

/METHOD=ENTER npaper televis socmdia

/METHOD=ENTER SixtyCd LWTCd

/METHOD=ENTER sixty_int LWT_int

/PRINT=GOODFIT CI(95).

*DV: Perceived knowledge.

REGRESSION

/DESCRIPTIVES MEAN STDDEV CORR SIG N

/MISSING PAIRWISE

/STATISTICS COEFF OUTS CI(95) R ANOVA CHANGE COLLIN TOL CHANGE ZPP

/CRITERIA=PIN(.05) POUT(.10)

/NOORIGIN

/DEPENDENT percknow

/METHOD=ENTER age male educ

/METHOD=ENTER npaper televis socmdia

/METHOD=ENTER sciint

/METHOD=ENTER SixtyCD LWTCd

/METHOD=ENTER sixty_int sciint LWT_int

/CASEWISE PLOT(ZRESID) OUTLIERS(3).

*/RESIDUALS DURBIN HISTOGRAM(ZRESID) NORMPROB(ZRESID)

*testing significant differences across groups - NS .23

UNIANOVA easy BY SixtyCd LWTCd InaCondition

/METHOD=SSTYPE(3)

/INTERCEPT=INCLUDE

/POSTHOC= SixtyCd LWTCd InaCondition (TUKEY).

CROSSTABS

/TABLES=LWTCd SixtyCd InaCondition BY Male

/FORMAT=AVALUE TABLES

/CELLS=COUNT

/COUNT ROUND CELL.

*Descriptives by condition

* SORT CASES BY InaCondition.

* SPLIT FILE LAYERED BY InaCondition.

* DESCRIPTIVES age educ sciint npaper televis socmdia.

**RESET

DESCRIPTIVES age education sciint.

DESCRIPTIVES npaper televis socmdia.

DESCRIPTIVES percknow.

FREQUENCIES gearti easy cures wideuse.

*Repeat for each condition

1. **SPSS Syntax for the supplementary materials.**

LOGISTIC REGRESSION VARIABLES gearti

/METHOD=ENTER sciint

/METHOD=ENTER SixtyCd LWTCd

/METHOD=ENTER sixty_int LWT_int

/SAVE=PRED COOK LEVER DEV SRESID

/PRINT=GOODFIT CI(95)

/CRITERIA=PIN(.05) POUT(.10) ITERATE(20) CUT(.5).

*DV: getting the "CRISPR is easy" question correct (no demographics no media).

LOGISTIC REGRESSION VARIABLES easy

/METHOD=ENTER sciint

/METHOD=ENTER SixtyCd LWTCd

/METHOD=ENTER sixty_int LWT_int

/PRINT=GOODFIT CI(95)

/CRITERIA=PIN(.05) POUT(.10) ITERATE(20) CUT(.5).

**Last Week Tonight (PROCESS OUTPUT)

DATA LIST FREE/
   LWTCd      sciint     easy       prob       .
BEGIN DATA.
        .00       1.19       1.84        .86
       1.00       1.19       2.71        .94
        .00       2.07       2.17        .90
       1.00       2.07       2.19        .90
        .00       2.95       2.51        .92
       1.00       2.95       1.68        .84
END DATA.

**60 Minutes (PROCESS OUTPUT)

DATA LIST FREE/
   SixtyCd    sciint     easy       prob       .
BEGIN DATA.
        .00       1.19       1.81        .86
       1.00       1.19       2.48        .92
        .00       2.07       1.83        .86
       1.00       2.07       2.58        .93
        .00       2.95       1.86        .87
       1.00       2.95       2.69        .94
END DATA.

*DV: getting the "cures" question correct.

LOGISTIC REGRESSION VARIABLES cure

/METHOD=ENTER sciint

/METHOD=ENTER SixtyCd LWTCd

/METHOD=ENTER sixty_int LWT_int

/PRINT=GOODFIT CI(95)

/CRITERIA=PIN(.05) POUT(.10) ITERATE(20) CUT(.5).

*DV: getting the "wide use" question correct.

LOGISTIC REGRESSION VARIABLES wideuse

/METHOD=ENTER sciint

/METHOD=ENTER SixtyCd LWTCd

/METHOD=ENTER sixty_int LWT_int

/PRINT=GOODFIT CI(95).

REGRESSION

/DESCRIPTIVES MEAN STDDEV CORR SIG N

/MISSING PAIRWISE

/STATISTICS COEFF OUTS CI(95) R ANOVA CHANGE COLLIN TOL CHANGE ZPP

/CRITERIA=PIN(.05) POUT(.10)

/NOORIGIN

/DEPENDENT percknow

/METHOD=ENTER sciint

/METHOD=ENTER SixtyCD LWTCd

/METHOD=ENTER sixty_int sciint LWT_int .
