# Supplementary material for: CRISPR is easy: Exposure to Last Week Tonight enhances knowledge about gene editing
Source: PLoS One. 2024 Oct 4;19(10):e0306563. doi: 10.1371/journal.pone.0306563 (PMC11452040; doi:10.1371/journal.pone.0306563)
Supplement: S3 File — (DOCX) [file pone.0306563.s003.docx]

**Supplementary models for CRISPR is easy: Exposure to Last Week Tonight enhances knowledge about gene editing, PLoS, June 2024**

**Supplementary table 1: Logistic regression analyses for the probability of choosing the gene editing article.**

| **Block** | **Odds ratio** | **95% CI** | **Sig.** |
| --- | --- | --- | --- |
| **1: Interest** |  |  |  |
| **Interest in scientific research and discoveries** | 1.18 | .91, 1.52 | .21 |
| **R^2^** | .7% |  |  |
| **2: Conditions** |  |  |  |
| **Sixty Minutes** | 1.27 | .73, 2.23 | .46 |
| **Last Week Tonight** | 1.10 | .63, 1.93 | .73 |
| **R^2^** | 1.0% |  |  |
| **3: Interactions** |  |  |  |
| **60 Minutes*interest** | .93 | .52, 1.76 | .88 |
| **Last Week Tonight*interest** | .87 | .45, 1.65 | .45 |
| **R^2^** | 1.1% |  |  |
| ***N*** | 303 |  |  |
| ***N chose article*** | 158 |  |  |
| ***df*** | 7 |  |  |
| **X^2^** | 2.4 |  | .78 |

Degrees of freedom and X^2^ are from the final model. R^2^ is Nagelkerke R^2^.

**Supplementary table 2: Probability of choosing the correct answer from the clip.**

| **Block** | **Easy** | | | **Cures** | | | **Wide Use** | | |
| --- | --- | --- | --- | --- | --- | --- | --- | --- | --- |
|  | **Odds** | **95% CI** | **Sig.** | **Odds** | **95% CI** | **Sig.** | **Odds** | **95% CI** | **Sig.** |
| **1: Interest** |  |  |  |  |  |  |  |  |  |
| **Interest** | 1.06 | .70, 1.61 | .79 | 1.93 | 1.15, 3.24 | .01 | 1.26 | .89, 1.78 | .19 |
| **R^2^** | .1% |  |  | 5.7% |  |  | 1.0% |  |  |
| **2: Cond.** |  |  |  |  |  |  |  |  |  |
| **60 Min.** | 2.11 | .78, 5.72 | .14 | 3.86 | 1.16, 12.82 | .03 | 2.36 | .1.05, 5.32 | .04 |
| **LWT** | .98 | .42, 2.31 | .97 | 3.78 | 1.14, 12.55 | .03 | 1.35 | .65, 2.78 | .42 |
| **R^2^** | 2.3% |  |  | 12.8% |  |  | 3.7% |  |  |
| **3: Inter.** |  |  |  |  |  |  |  |  |  |
| **60 Min.*Int.** | .64 | .22, 1.90 | .42 | 1.31 | .37, 4.55 | .68 | 1.12 | .47, 2.66 | .79 |
| **LWT*Int.** | .32 | .11, .91 | .03 | 1.66 | .41, 6.81 | .48 | .63 | .27, 1.47 | .28 |
| **R^2^** | 5.7% |  |  | 17.1% |  |  | 4.7% |  |  |
| ***N*** | 281 |  |  | 278 |  |  | 280 |  |  |
| ***N correct*** | 249 |  |  | 259 |  |  | 232 |  |  |
| ***df*** | 7 |  |  | 7 |  |  | 7 |  |  |
| **X^2^** | 8.25 |  | .14 | 14.29 |  | .01 | 8.05 |  | .15 |

Degrees of freedom and X^2^ are from the final model. R^2^ is Nagelkerke R^2^.

Given the result of the first model, we used the SPSS PROCESS macro [1] model 1 to probe the significant interaction using the Johnson-Neyman technique. We specified that Y was the “easy” question, X was Last Week Tonight, and the moderator (W) was interest. The other variable from the model was included as a covariate. Though the results showed that the interaction findings were not significant by conventional standards, there is evidence, if modest, that at the lowest level of interest (0 level and .14 levels of interest, p = .08), information-laden entertainment content can exert an equalizing effect, producing expected values for low and high interest participants that are effectively indistinguishable.

We again used SPSS PROCESS macro model 1 to compare *Last Week Tonight* to control and to plot *Last Week Tonight* compared only to the control condition. Levels of interest were conditioned on -1 standard deviation (low interest), +1 standard deviation (high interest) from the mean (medium interest). Comparing the control condition and *Last Week Tonight*, at the lowest level of interest, there was a .86 and .94 probability of answering the question correctly, respectively. Notably, comparing the control condition and the *Last Week Tonight* condition at the highest levels of interest, there was a .92 and .85 probability, respectively. See Supplementary Figure 1.

**Supplementary Fig. 1: Last Week Tonight compared to control regarding the correct answer to CRISPR’s ease of use.
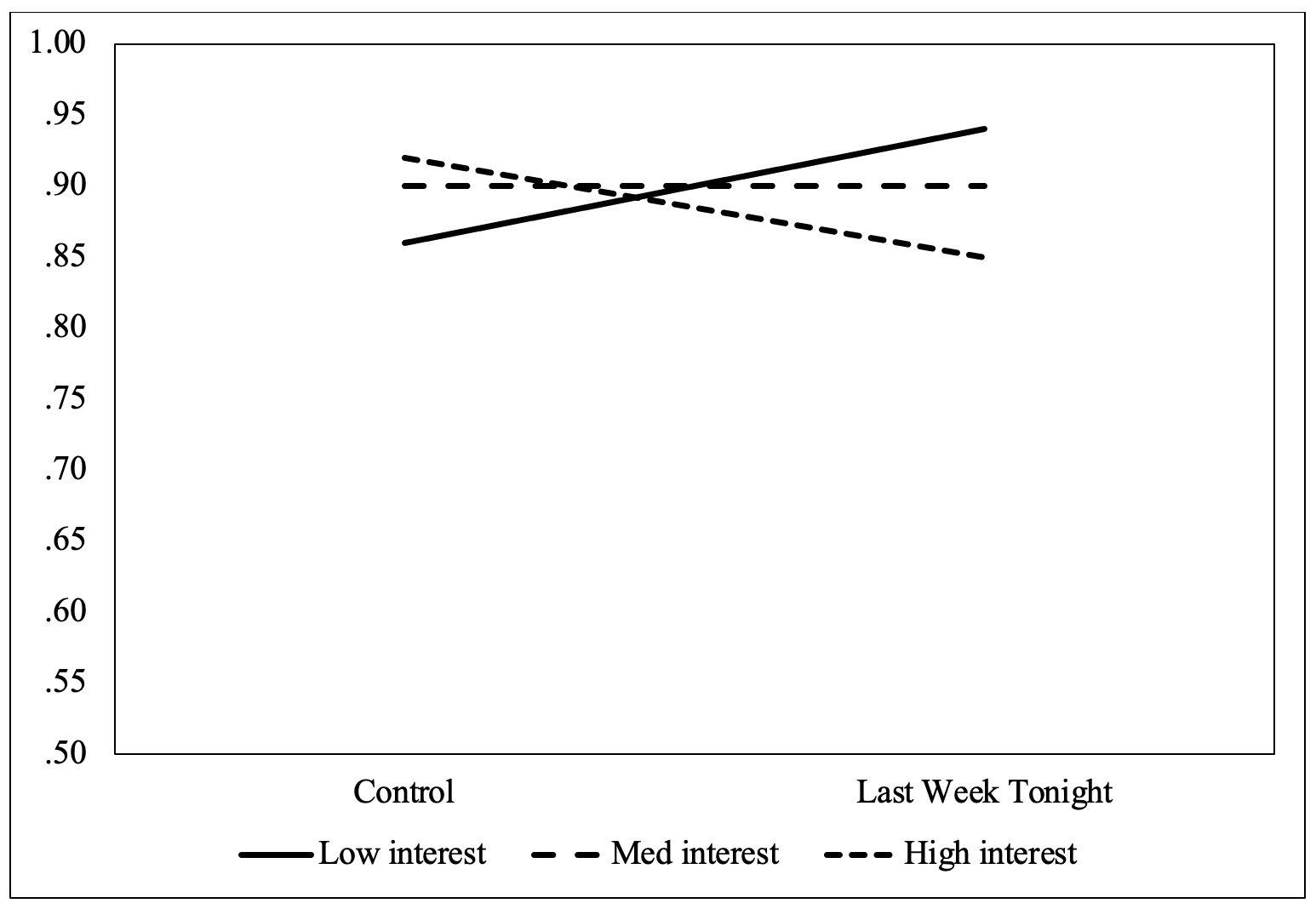
**

Though we did not find a significant result for the 60 Minutes condition, we offer it to show a comparison. Using the same specifications as above but X was specified as *60 Minutes*. Comparing the control condition and *60 Minutes*, at the lowest level of interest, there was a .86 and .92 probability of answering the question correctly, respectively.

**Supplementary Fig. 2: 60 Minutes compared to control regarding the correct answer to CRISPR’s ease of use.**
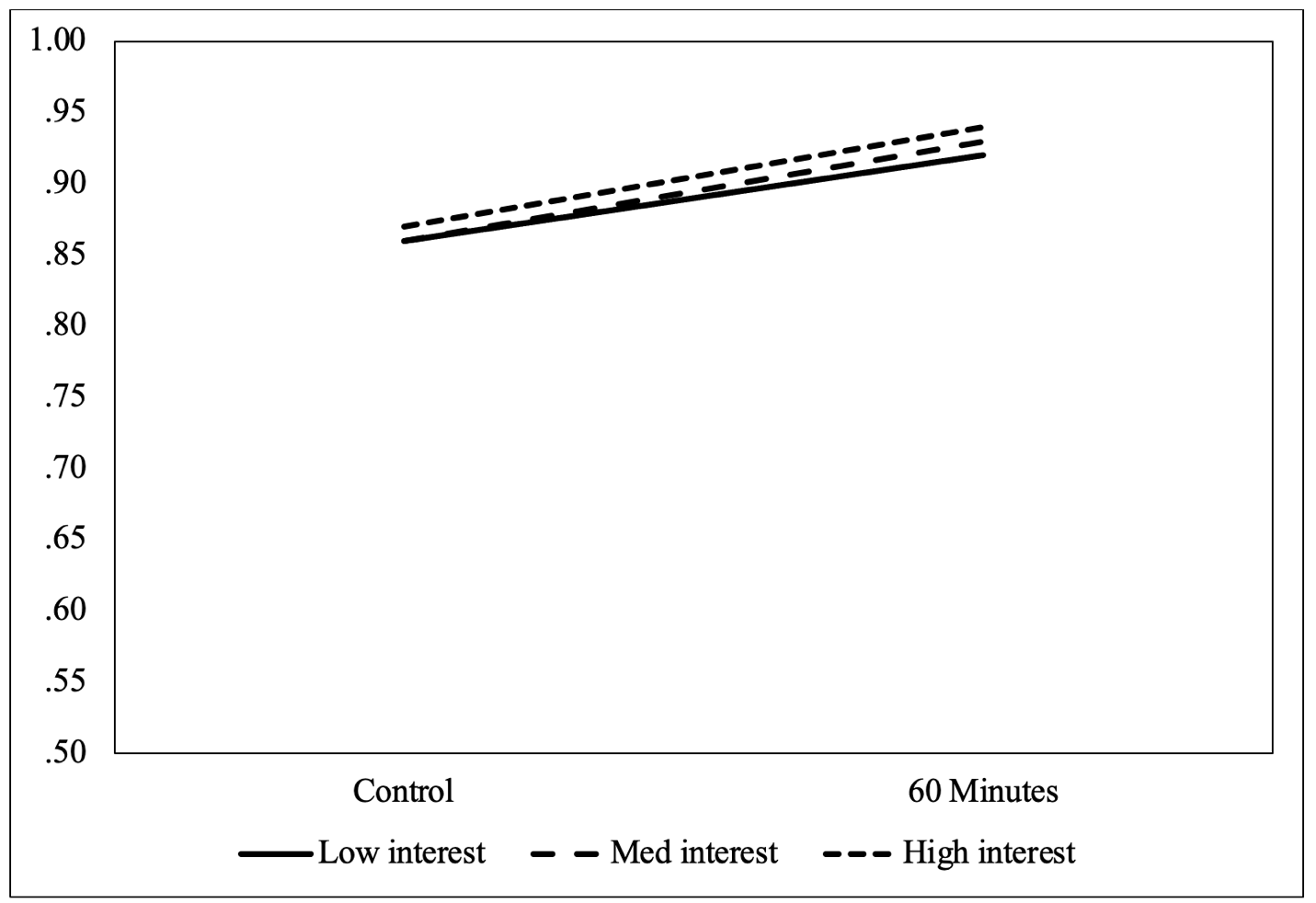


In the *Last Week Tonight* condition, participants with the lowest levels of science interest showed distinct gains in knowledge from exposure to the condition, whereas there seem to be, if anything, negative gains from exposure for those with higher levels of interest.

**Supplementary table 3: Regression analyses showing entries by block for perceived knowledge.**

|  | **B** | **95% CI** | **SE** | **β** | **Sig.** |
| --- | --- | --- | --- | --- | --- |
| **1: Interest** |  |  |  |  |  |
| **Interest in scientific research and discoveries** | .16 | .06, .27 | .05 | .18 | <.001 |
| **Inc. Adjusted R^2^ (%)** | 3.4 % |  |  |  |  |
| **R^2^ Change** | 3.7 |  |  |  | <.001 |
| **2: Conditions** |  |  |  |  |  |
| **Sixty Minutes** | .52 | .30, .73 | .11 | .30 | <.001 |
| **Last Week Tonight** | .40 | .18, .61 | .11 | .23 | <.001 |
| **Inc. Adjusted R^2^ (%)** | 10.0% |  |  |  |  |
| **R^2^ Change** | 7.2 |  |  |  | <.001 |
| **3: Interactions** |  |  |  |  |  |
| **60 Minutes*Interest** |  |  |  | .04 | .81 |
| **Last Week Tonight*Interest** |  |  |  | .18 | .24 |
| **Inc. Adjusted R^2^ (%)** | 10.0% |  |  |  | .34 |
| **R^2^ Change** | .6 |  |  |  |  |
| **N = 303** |  |  |  |  |  |
| **Total *R*^2^ (%)** | 11.5% |  |  |  |  |

Reference:

1. Hayes AF. Introduction to mediation, moderation, and conditional process analysis: A regression-based approach: Guilford publications; 2017.
